# Exploring the relationship between premarital sex and cigarette/alcohol use among college students in Taiwan: a cohort study

**DOI:** 10.1186/1471-2458-12-527

**Published:** 2012-07-18

**Authors:** Chi Chiao, Chin-Chun Yi, Kate Ksobiech

**Affiliations:** 1 Insitute of Health and Welfare Policy, Research Center for Health and Welfare Policy, School of Medicine, National Yang-Ming University, Taipei, Taiwan, R.O.C; 2 Institute of Sociology, Academia Sinica, Taipei, Taiwan, ROC; 3 Communication Department, College of Arts and Communication, University of Wisconsin-Whitewater, Whitewater, WI, USA

**Keywords:** Premarital sex, Adolescent cigarette and alcohol use, Peer influence, College students, Taiwan

## Abstract

**Background:**

Cigarette/alcohol use and premarital sex, and their subsequent consequences on the well-being of college students, are international health promotion issues. However, little is known about the temporal relationship of these risk behaviors among Taiwanese college students.

**Methods:**

This study utilizes data from the Taiwan Youth Project, a cohort sample of 20-year-olds (N = 2,119) with a 2-year follow-up, to explore the relationship between adolescent cigarette/alcohol use, and subsequent premarital sex. To incorporate the Taiwanese context where the normative value of abstinence until marriage remains strong, multivariate logistic regression models included data on premarital sex attitudes, stressful life events, peer influence, as well as family and individual factors which might influence this relationship.

**Results:**

The sample consists of 49% male and 51% female college students. About 16% of the sample report having had premarital sex by age 20. After excluding sexually active youth, 20% of males and 13% of females report engaging in premarital sex in the 2-year follow-up interview. Multivariate logistic regression analyses reveal adolescent alcohol use is significantly associated with a higher likelihood of engaging in premarital sex for both genders; adolescent smoking is significantly associated with premarital sexual activity among males, but not females. Our results indicate liberal premarital sexual attitudes and stressful personal events are also significantly associated with premarital sexual activity.

**Conclusions:**

These findings suggest health promotion programs for college students need to take developmental and gender perspectives into account. Future research to incorporate a broader, multi-cultural context into risk reduction materials is recommended.

## Background

Risky health behaviors such as cigarette/alcohol use, as well as the onset of sexual activity, are common from late adolescence to young adulthood in East Asian countries such as Taiwan. These behaviors increase an individual’s subsequent mental and physical health risks to some degree [1-4]. Studies indicate nearly one-quarter of male youth in Taiwan self-report cigarette use at least once by the end of high-school, and the smoking rate more than doubles to 48% by the end of college [5]. Although female cigarette use was somewhat lower among Taiwanese high school students (4-5%), smoking prevalence, particularly for youth, has continued to rise since Taiwan opened its tobacco market to foreign companies in 1987 [6]. A recent population-based survey in Taiwan reports alcohol use by age 18 at about 38% [1]; not surprisingly, that percentage is predicted to increase as adolescents move into early adulthood [7]. Similarly, the same study [7] reports premarital sex by age 18 among female adolescents in Taiwan is 22% among 15–19 year-old cohorts, but only 17% among those 20–24 year-old cohorts. Social norms regarding premarital sex are apparently becoming more liberal in recent times.

Problem behaviors (e.g., smoking, drinking, drug use, premarital sex) tend to cluster in youth as distinct social behaviors [2,8-11]. If the relationship between cigarette/alcohol use and subsequent sexual activity can be understood, interventions can be designed to efficiently and effectively decrease and/or limit the likelihood of adolescents’ misusing these substances and engaging in premarital sex. Thus far, there is relatively little empirical evidence regarding this link in Asian countries like Taiwan.

Mason and colleagues [12] analyzed data gathered from a sample of 808 American youth, followed from age 10 to age 24. Their findings suggest early alcohol use correlates with a higher likelihood of risky sex. However, that study is based on a Western population, and its analyses did not include factors related to social context and peer influence; therefore, additional risk behaviors often associated with adolescent development were not assessed.

Using the Theory of Planned Behavior [13-15] and Social Learning Theory [16,17], this study explores the association between adolescent cigarette/alcohol use and premarital sex from a temporal perspective. The Theory of Planned Behavior asserts an adolescent’s intention to engage in premarital sex flows from a youth’s attitude toward premarital sex, his/her perception of the subjective (cultural) norms associated with such behavior, and his/her beliefs regarding his/her ability to engage in sexual activity. How these factors are influenced by relationships with peers and stressful life events are also central to the dynamics of when, how, and to what degree youth engage in premarital sex. In addition, Social Learning Theory posits peer group interaction portrays a significant role in cigarette/alcohol use and subsequent premarital sex among college students. In a sample of Taiwanese youth who successfully completed a competitive college entrance exam, adolescents report peer pressure as a powerful influence in their decision to initiate and experiment with a variety of risky health behaviors [16,18]. Previous research in the Philippines and Taiwan identifies perceived sexual activity by best friends as an influential factor in the initiation of sexual activity in an adolescent’s own life [8,19]. In the present cohort study, we extend our analyses to include the temporal relationship between peer pressure and premarital sex during college.

Using longitudinal data from the Taiwan Youth Project, we specifically address this research question: What is the relationship between adolescent cigarette/alcohol use and the likelihood of premarital sex during college, after controlling for premarital sex attitudes, peer influence, life and behavioral events, and family background? The analyses were conducted separately for male and female college youth, due to gender differences suggested by a wide range of existing literature [7,19].

Taiwan provides an interesting cultural context to investigate this question. Traditional Taiwanese culture values conservative social norms such as responsibility, restraint, interdependency and collectivism, suggesting premarital sex is not endorsed, especially among single adults [20]. Taiwanese college students living in this cultural context are exposed to a high degree of normative and institutional influence, which leads them to be “well-behaved” and not engage in smoking, drinking, or premarital sex in late adolescence. Over three-fourths of Taiwanese high school graduates enroll in college [21,22]. Studies have shown the school environment serves as a protective factor leading to less deviance, less psychological maladaptation and less premarital sex in Taiwan [8,23]. Even within this context, however, risky behaviors such as cigarette/alcohol use and premarital sex occur, creating a salient public health issue deserving further investigation.

## Methods

### The study population

The Taiwan Youth Project (TYP) was launched in 2000 and originally included 5,541 junior high students (2,740 girls and 2,801 boys) in northern Taiwan (Taipei city, Taipei county and Yi-Lan county) as the baseline with an annual follow-up through self-administered questionnaires and telephone interviews. Data on the sensitive topics of sexual and intimate activities and attitudes were gathered in this cohort since 2004 when youth turned 20 years old [8]. In order to understand sexual behavior after age 20, and to hold constant the documented protective context of being in school, our analytic sample is restricted to never-married college youth, surveyed at age 20 and again at age 22, with complete data on self-reported premarital sex, premarital sexual permissiveness attitudes and other major covariates discussed herein. The inclusion criteria yielded a total study sample of 2,119 college youth.

### Measures

#### The dependent variable

*Premarital sex involvement* was measured by a self-reported question: “Have you ever had penile-vaginal intercourse?” with a dichotomous response (i.e., “yes” or “no”). This sexual activity item was included in questionnaires for youth at both ages 20 and 22.

#### Explanatory variables

Late adolescence (18–21 years old) characterizes the peak of sensation seeking associated with an increased likelihood of engaging in physical and social risks, such as risky health behaviors [24]. We defined respondents’ cigarette and alcohol use by age 20 as *adolescent risky health behaviors*.

*Adolescent cigarette and alcohol use* was defined by whether never-married 20 year-old college youth self-reported cigarette and/or alcohol use. This information was obtained directly from the questionnaire item, “Have you smoked [cigarettes] in the past week?” and, “Have you drunk [alcohol] in the past month?” Respondents were categorized as “smokers” and “non-smokers.” For the alcohol use item, students were placed in one of three groups: “abstainer” (no alcohol use), “light drinker” (1–2 times per month) and “heavy drinker” (3+ times per month).

*Peer characteristics* included respondent perceptions of three behaviors: perceived sexual behavior of close friends, perceived cigarette use of close friends, and perceived alcohol use of close friends. Respondents were asked about the perceived sexual experiences of their close friends with possible responses being “none, less than half, and half or greater” of their close friends having had premarital sex. They were also asked whether their close friends smoked and the frequency of alcohol use among their close friends. Smoking responses were dichotomized (“smoker” and “non-smoker”) for friends perceived smoking behavior, and close friends’ perceived alcohol use responses were placed into 1 of 3 categories (“abstainer,” “light drinker = 1–2 times in past month,” and “heavy drinker = 3 or more times in past month”).

*Stressful life, school and behavioral events* included 4 major types: a stressful school event within the previous 12 months, a stressful personal event occurring during the previous 12 months, deviant behavior within the previous 12 months, and/or psychological distress. Each stressful school event was dichotomized (0 = no; 1 = yes), indicating whether they had experienced any one of four “stressful” school events: 1) withdrawal from school, 2) not getting along with classmates, 3) took a health-related leave of absence from school, or, 4) had a behavioral problem at school. Participants also reported whether they had experienced one or more of the following personal stressful life events in the past year: death of a parent, death of a close relative, death of a pet, or ending a relationship with a girlfriend or boyfriend. We categorized the number of stressful personal events experienced into 3 levels: 0, 1, and 2 or more events. The indicator of the “deviant behavior” independent variable was determined by a self-reported acknowledgement of participating in one or more of the following activities: using illegal drugs, stealing, cheating on exams, and destroying public property. In addition, psychological depression was assessed via the Symptom-Checklist-90-Revised (SCL-90-R; [24]). This scale contains 16 items assessing affective, cognitive, and vegetative dimensions of depression. The SCL-90-R Depression scale has been shown to be both reliable and valid [25], and has been used in a previous study of Taiwanese adolescents [23]. The total score ranged from 0 to 64 with Cronbach α at 0.90. Higher scores are associated with greater levels of depressive symptoms.

*Premarital sexual permissiveness* was measured by a 3-item scale asking youth to report their agreement levels regarding premarital *kissingpetting*, and *coitus* with a partner for whom they have strong affection. The response ranged from “strongly agree” to “strongly disagree” on a 6-point scale [26]. As prior research suggested [27], each permissiveness measure was dichotomized to “permissive” vs. “conservative” attitudes and the three items were summed to produce a permissiveness scale, with scores ranging from 0 to 3. Higher scores are associated with more permissive attitudes toward premarital intimate behaviors.

Several covariates were used as potential confounding variables affecting the relationships between the main explanatory variables and premarital sex involvement. *Perceived support* came from one or both of two sources: perceived friend support and perceived family support. In the TYP survey, respondents were asked to rate the availability of perceived support from both family and friends (separate items), in 3 areas: school work, economic strain, and personal emotion. Other covariates were *family socioeconomic status* measured by maternal education (3 categories: junior high school or lower, senior high school, and college or higher); family structure (living with both biological parents vs. other); and monthly family income with categorical responses.

### Statistical Analysis

To examine the temporal association between adolescent cigarette/alcohol use and sexual activity during college, we began with bivariate analyses that characterized the sample profile by sexually active college participants by age 20. We then used multivariate logistic regression models that elaborated and progressively adjusted the significance of respondent cigarette and alcohol use on later premarital sex in college. Model 1 elaborated adolescent cigarette and alcohol use to investigate our primary research interest. Model 2 added peer characteristics to test for possible confounding of our primary research question. Model 3 continued by including attitudes toward premarital sex, stressful life and behavioral events, and other covariates to determine adjusted effects of adolescent cigarette/alcohol use on subsequent premarital sex.

In these multivariate models, we limited the analysis to sex abstainers at age 20 who continued to be enrolled in college (N = 1,777). As mentioned above, males and females are studied separately to explore the potential gender specific associations. In analyses not shown here, we also tested for interactions between peer influence and adolescent cigarette/alcohol use variables, to examine whether these risky behaviors varied by perceived peer characteristics for each gender. However, because these interactions did not add significantly to the models, they are not included here. All logistic regression models, reported as an adjusted odds ratio (AOR), controlled for sample clustering effects. Missing data in the regressions used listwise deletion. Variables used in regression models may be highly correlated. We employed analyses of the variance inflation factor (VIF) to assess multicollinearity. VIFs less than 2 were not regarded as a multicollinearity concern. Statistical analysis was performed with STATA 11.0 [28].

## Results

### Descriptive findings

Table 1 summarizes sample characteristics stratified into two groups: participants who had or had not engaged in premarital sex by age 20. Overall, most characteristics differed markedly between these two groups, and by gender. For instance, sexually active youth were more likely to report cigarette/alcohol use than participants who were sexually abstinent. In each group, male students reported significantly more risky behaviors than female students. Sexually active youth were also more likely to believe their close friends smoked and drank and engaged in premarital sex, with significantly higher proportions among males than females in each group. In contrast, sexually abstinent youth were more conservative in their premarital sexual attitudes and experienced less stressful life and behavior events. In each group, females reported higher levels of stressful life and behavioral events than males. The percentage of sexually abstinent 20-year-old college students who engaged in premarital sex by age 22 was significantly higher among males (20%) than females (13%).

**Table 1:** Sample characteristics for male and female college youth (percentage or mean), TYP

**Covariates at age 20**	**TotalN = 2119**	**Premarital sex by age 20(n = 342)**		**No premarital sex by age 20(n = 1777)**	
		**Male**	**Female**	**Male**	**Female**
**Adolescent risky health behavior**					
Adolescent's cigarettes use					
Smokers	7.8	32.7	12.3	7.9	1.5
Non-smokers	92.2	67.3	87.7	92.1	98.5
Adolescent's alcohol use					
Heavier drinkers	4.2	16.1	5.4	3.8	1.8
Light drinkers	21.2	32.7	30.8	23.3	15.5
Abstainers	74.6	51.2	63.9	72.9	82.7
**Peers characteristics**					
Perceived cigarettes use of best friends					
Smokers	21.4	58.8	23.8	25.5	9.2
Non-smokers	78.6	41.2	76.2	74.5	90.8
Perceived alcohol use of best friends					
Heavier drinkers	5.5	19.4	7.7	5.3	2.3
Light drinkers	22.2	36.0	26.9	24.3	16.7
Abstainers	72.3	44.6	65.4	70.4	81.1
Perceived sexual behavior of best friends					
Half or greater	12.6	44.3	40.0	7.3	6.4
Less than half	27.3	34.9	36.9	26.3	25.2
None or unknown	60.1	20.8	23.1	66.3	68.4
**Attitude toward premarital sex**					
Premarital sex permissiveness, range 0–3, (mean)	2.5	3.0	2.9	2.6	2.2
**Stressful life and behavioral events**					
Stressful school events					
Yes	13.7	17.9	28.5	11.1	13.0
No	86.3	82.1	71.5	88.9	87.0
Number of stressful personal events					
2 or more	16.7	25.9	42.3	11.7	15.5
1	27.6	31.6	23.9	27.4	27.3
0	55.7	42.5	33.9	60.9	57.3
Deviant behaviors					
Yes	31.0	63.7	30.0	42.2	14.3
No	69.0	36.3	70.0	57.8	85.7
Psychological complaints (mean)	8.6	9.2	11.7	7.1	9.2
**Family SES**					
Maternal education					
Junior HS or lower	42.2	47.6	42.2	40.4	42.6
Senior HS	35.7	30.8	41.4	36.7	35.1
College or higher	22.1	21.6	16.4	22.8	22.4
Monthly family income					
<50000	35.2	30.3	38.6	33.3	37.6
50000-69999	29.7	26.9	26.0	32.3	28.7
70000 & above	35.1	42.8	35.4	34.5	33.8
Family structure: Lives with both biological parents					
Yes	80.8	72.2	61.5	82.0	84.2
No	19.3	27.8	38.5	18.1	15.8
**Perceived family support**					
School work					
Yes	64.3	57.6	55.4	60.1	70.6
No	35.7	42.5	44.6	39.9	29.4
Economic difficulty					
Yes	87.3	84.0	83.1	85.6	90.0
No	12.7	16.0	16.9	14.4	10.0
Emotional difficulty					
Yes	44.6	31.6	50.0	38.4	52.1
No	55.4	68.4	50.0	61.6	47.9
**Perceived friend support**					
School work					
Yes	79.9	82.1	80.0	74.8	83.8
No	20.1	17.9	20.0	25.2	16.2
Economic difficulty					
Yes	25.4	36.3	46.2	24.2	21.2
No	74.6	63.7	53.9	75.9	78.8
Emotional difficulty					
Yes	85.1	91.0	93.9	78.7	88.2
No	14.9	9.0	6.2	21.3	11.8
**Premarital sex in college**					
Involvement in premarital sex by age 22					
Yes	28.1	--	--	20.2	13.1
No	71.9	--	--	79.8	86.9

*Note*: Participants who missed responses on covariates were excluded. Percentages may not add up to 100 due to rounding.

### Relationship between premarital sex and adolescent cigarette/alcohol use

Table 2 presents logistic regression models to investigate the effects of adolescent cigarette/alcohol use, perceived peer characteristics, stressful life and behavioral events, and other adolescent covariates on later premarital sexual behavior by gender. In order to *predict* premarital sex during college, the multivariate analyses focused on the sexually abstinent youth at age 20. Model 1 shows adolescent cigarette and alcohol use increased the likelihood of engaging in premarital sex. Adolescent cigarette use was significantly related to engaging in premarital sex for males only (AOR = 2.4; 95% CI = 1.4-4.0) while adolescent alcohol was significantly related to engaging in premarital sex for females only (AOR = 2.0; 95% CI = 1.4-4.0). Model 2 adds peer characteristics. Adolescent cigarette use was still significantly associated with higher odds of premarital sex for males only (AOR = 2.0; 95% CI = 1.4-4.0). However, adolescent alcohol use also appeared to be a significant predictor of premarital sex for males and females when peer characteristics were controlled.

**Table 2:** Results from multivariate logistic regression models of predicting involvement in premarital sex by age 22, TYP (N=1,777)

	**Male**						**Female**					
	**Model 1**		**Model 2**		**Model 3**		**Model 1**		**Model 2**		**Model 3**	
*Covariates at age 20*	AOR	95% CI	AOR	95% CI	AOR	95% CI	AOR	95% CI	AOR	95% CI	AOR	95% CI
**Adolescent risky health behavior**												
Adolescent's cigarettes use												
Smokers	2.4^**^	(1.4-4.0)	2.0^**^	(1.2-3.5)	2.1^*^	(1.2-3.9)	2.1	(0.7-6.5)	0.9	(0.3-3.3)	1.0	(0.3-3.7)
Non-smokers	1.0		1.0		1.0		1.0		1.0		1.0	
Adolescent's alcohol use												
Heavier drinkers	2.0	(0.9-4.4)	2.7^§^	(1.0-7.5)	2.7^*^	(1.1-6.8)	3.0^*^	(1.1-8.4)	2.0	(0.7-6.0)	2.0	(0.7-5.9)
Light drinkers	1.2	(0.8-1.9)	1.4	(0.9-2.3)	1.2	(0.8-2.1)	2.4^***^	(1.5-3.8)	2.4^**^	(1.4-4.2)	2.1^*^	(1.2-3.6)
Abstainers	1.0		1.0		1.0		1.0		1.0		1.0	
**Peers characteristics**												
Perceived cigarettes use of close friends												
Smokers			0.9	(0.5-1.5)	0.9	(0.5-1.4)			3.0^***^	(1.6-5.5)	2.4^**^	(1.3-4.7)
Non-smokers			1.0		1.0				1.0		1.0	
Perceived alcohol use of close friends												
Heavier drinkers			0.5	(0.2-1.4)	0.5	(0.2-1.3)			0.9	(0.4-1.3)	1.1	(0.3-4.4)
Light drinkers			0.7	(0.4-1.2)	0.7	(0.4-1.2)			0.7	(0.2-3.5)	0.7	(0.4-1.2)
Abstainers			1.0		1.0				1.0		1.0	
Perceived sexual behavior of close friends												
Half or greater			3.5^***^	(1.9-6.5)	3.1^**^	(1.6-6.1)			2.5^*^	(1.2-5.2)	1.8	(0.8-4.0)
Less than half			1.4^§^	(0.9-2.2)	1.4	(0.9-2.1)			1.6^*^	(1.03-2.5)	1.2	(0.7-2.1)
None or unknown			1.0		1.0				1.0		1.0	
**Attitude toward premarital sex**												
Premarital sex permissiveness					1.3^§^	(0.97-1.9)					1.6^**^	(1.2-2.3)
**Stressful life and behavioral events**												
Stressful school events												
Yes					0.6	(0.3-1.1)					0.9	(0.5-1.5)
No					1.0						1.0	
Number of stressful personal events												
2 or more					2.7^***^	(1.6-4.7)					1.7^*^	(1.001-3.0)
1					1.4^§^	(0.96-2.1)					1.6^§^	(0.995-2.5)
0					1.0						1.0	
Deviant behaviors												
Yes					1.0	(0.7-1.4)					1.1	(0.6-1.8)
No					1.0						1.0	
Psychological complaints					1.0	(1.0-1.0)					1.0	(1.0-1.0)
**Family SES**												
Maternal education												
College or higher					0.6^*^	(0.3-0.99)					0.7	(0.4-1.1)
Senior HS					0.9	(0.6-1.4)					0.9	(0.6-1.3)
Junior HS or lower					1.0						1.0	
Monthly family income					1.1	(1.0-1.1)					1.1^*^	(1.004-1.2)
Family structure												
Lives with both biological parents					1.0	(0.6-1.6)					0.7	(0.4-1.1)
Others					1.0						1.0	
**Perceived family support**												
School work												
Yes					1.3	(0.8-2.0)					1.1	(0.7-1.9)
No					1.0						1.0	
Economic difficulty												
Yes					0.8	(0.5-1.3)					1.1	(0.5-2.3)
No					1.0						1.0	
Emotional difficulty												
Yes					0.8	(0.5-1.2)					0.8	(0.5-1.2)
No					1.0						1.0	
**Perceived friend support**												
School work												
Yes					0.9	(0.6-1.4)					2.9^*^	(1.3-6.6)
No					1.0						1.0	
Economic difficulty												
Yes					0.9	(0.6-1.4)					1.1	(0.7-1.8)
No					1.0						1.0	
Emotional difficulty												
Yes					2.0^*^	(1.1-3.5)					0.8	(0.4-1.6)
No					1.0						1.0	

*Note*: All logistic regression models adjust for sampling cluster; AOR represents adjusted odds ratios for sample cluster; CI represents confidence interval; ^§^p < 0.10, ^*^p < 0.05, ^**^p < 0.01, ^***^p < 0.001.

Model 3 progressively adds variables measuring attitudes toward premarital sex, stressful life and behavioral events and other individual/family characteristics to Model 2. Even after controlling for other covariates, adolescent cigarette use remained significant in predicting premarital sex for male college students only (AOR = 2.1; 95% CI = 1.2-3.9). In addition, male college students with heavier adolescent drinking were 2.7 times more likely to have premarital sex than abstainers; this result continued to be found among females categorized as light drinkers (AOR = 2.1; 95% CI = 1.2-3.6).

For males, most peer effects were not significantly related to premarital sex; the only exception was the male’s perception of close friends’ sexual behavior. Male college students who perceived half or more of their close friends were sexually active by age 20 had a higher odds of having later premarital sex than those male respondents whose close friends were believed to be sexually abstinent, or if they were unaware of their close friends’ sexual activities (AOR = 3.1; 95% CI = 1.6-6.1). Experiencing stressful personal life events was significantly associated with higher odds of premarital sex during college for both sexes (AOR = 2.7; 95% CI = 1.6-4.7 for males and AOR = 1.7; 95% CI = 1.001-3.0 for females). With other factors controlled, multivariate analyses revealed college students with “liberal sexual permissiveness attitudes” were significantly more likely to engage in premarital sex (AOR = 1.3; 95% CI = 0.97-1.9 for males and AOR = 1.6; 95% CI = 1.2-2.3 for females).

After adjusting for other covariates, specific family factors associated with a significantly lower likelihood of premarital sex were: having a more highly-educated mother for male college students (AOR = 0.6; 95% CI = 0.3-0.996); and having a lower family income for female college students (AOR = 0.9; 95% CI = 0.9-0.996). Unexpectedly, significant risk factors for premarital sex during college were “perceived friend support for schoolwork” among females (AOR = 2.9; 95% CI = 1.3-6.6), and “perceived friend support for emotional difficulties” among males (AOR = 2.0; 95% CI = 1.1-3.5).

## Discussion

As theories and prior research suggest, our results found cigarette smoking, alcohol use, and premarital sex tend to cluster in youth [2,8-11]. Moreover, our findings demonstrated the influence of adolescent cigarette/alcohol use on later premarital sex, and showed gender differences related to self-reported premarital sex during college. Overall, adolescent cigarette/alcohol use was higher among Taiwanese youth who were sexually active by age 20 when compared to their sexually abstinent counterparts. In addition, for those abstinent youth, the *predictive* effect of adolescent cigarette and alcohol use differed for male and female. For males, adolescent “heavier drinking” was significantly related to later premarital sex (by age 22); however, for females, adolescent “light drinking” was significantly associated with later premarital sex. These results are consistent with previous findings of a positive association between alcohol use and premarital sexual activity [12,29,30]. Our analyses further suggest gender differences in a dose–response effect in which the likelihood of later premarital sex is associated with heavier drinkers in males vs. light drinkers in females. And, these results imply that females may suffer more than males from being alcoholic in social norms. The finding of no significant predictive effect of cigarette smoking on self-reported premarital sex for females is consistent with previous reports [31,32], but the significant predictive effect of cigarette smoking for males differs from the existing literature. This might be because the adolescent smoking rate is much lower among females in the sample (2%) than for males (8%). It is recommended future research studies examine how/if adolescent cigarette use links to female sexual activities.

Our findings are consistent with the Theory of Planned Behavior and its emphasis on cultural norms and attitudes as related to attitudes toward premarital sex. Previous studies found adolescent premarital sexual activity to be shaped by or associated with contextual factors [8,33-35]. These contextual factors (e.g., school attendance and community participation) impose conventional behavioral expectations with adolescents expected to abstain from sex until marriage. Prior research also showed a significant association between liberal attitudes toward premarital sex and premarital sexual activity in Nepal college males [36]. In our college sample, those adolescents with permissive premarital sexual attitudes were more likely to engage in premarital sex during college, regardless of gender. Premarital sex appears to be increasingly common in the romantic relationships of the Taiwanese young populace [27]. Our results suggest a cultural shift regarding premarital sexual activity among Taiwanese college-age youth. The increased acceptance of more liberal and permissive sexual attitudes is associated with the increased likelihood of sexual activity among Taiwanese youth. It is recommended that future cigarette, alcohol and sexual risk materials developed for Taiwanese college students take these shifting social norms into consideration and pay special attention to gender specific measures.

In addition, there are potentially serious health and social implications for those engaging in premarital sex without protection such as use of condoms or other contraceptives to prevent unwanted pregnancy or sexually transmitted diseases [37]. In fact, contraceptive use is not common among Taiwanese college students, although the proportion using contraceptives does increase by age [7]. In separate analyses, we found that less than half (43%) of college students who had premarital sex by age 20 reported consistent contraceptive use. Approximately half (52%) of college students who had premarital sex by age 22 self-reported consistent contraceptive use. Because premarital sex among college youth will likely become more prevalent, encouraging consistent contraceptive use should become a major point of emphasis for health professionals dealing with Taiwanese college students.

As suggested by Social Learning Theory, peer group influence plays a significant role in cigarette smoking, alcohol use, and premarital sex among college students. Our results partly supported that respondents’ perceptions of their close friends’ risky behaviors (cigarette smoking, alcohol use and sexual activity) were higher among youth who were sexually active by age 20 than those who were sexually abstinent. Even when controlling for adolescent risky health behaviors, multivariate analyses in Model 2 showed later premarital sex (by age 22) was positively associated with perceived close friends’ sexual behavior [8,19,36]. In contrast, the non-significant predictive effect of perceived cigarette smoking of best friends for males and non-significant predictive effect of perceived alcohol use of best friends for both sexes may be due to sample selection. Our college sample remained in the protective school environment throughout the time period of this study, a factor that may contribute to a delay in engaging in premarital sex. This finding might be interpreted via the *age-integrated* hypothesis [38], in which an increasing strength of social bond is particularly influential to college students who remained sexually abstinent by age 20. This bond to a social institution thus serves to mitigate peer influence as well as reduce problem behaviors.

This study addressed a seriously under-explored subject with regard to whether stressful life events are associated with the link between adolescent cigarette/alcohol use and premarital sex [39]. As prior research suggests, individuals may engage in premarital sex to escape from stressful life events, and such an effect may last as long as 2 years [40]. Consistent with the previous study using a U.S. sample [39], our findings demonstrated experiencing a stressful personal event was associated with a higher likelihood of engaging in premarital sex during college for both sexes in Taiwan. In addition, considerable evidence suggests an association between stressful life events and psychological distress in adolescence. Our findings further support this relationship. Additional research is needed to examine the relationships between and among stressful life events, initial sexual encounters, and long-term psychological well-being.

Consistent with prior research [8,33,36], family characteristics were found to be significantly related to later premarital sex among college students in Taiwan, but the results slightly differ for males versus females. The analyses suggest male college students with an educated mother were less likely to have premarital sex between 20 and 22; this relationship was not observed for female college students. In contrast, level of family income was inversely associated with increased odds of premarital sex involvement between 20 and 22 for female college students, but not for male college students. We suspect the differential gender effects on premarital sex may be due to the correlation between family SES and parenting in the college sample. While resourceful family reveals protective function, lower SES background tends to perform less parental control. Additional research is thus needed to compare the family SES context between males and females, and disentangle the various effects including parenting. Given families appear to be particularly influential for Taiwanese college students, understanding the ways in which *family* impacts sexual behavior is essential.

There are two major limitations in this study. Firstly, endogeneity may be present in the relationship between substance misuse and sexual activity [41]. Hence, we adopted a cohort sample to disentangle simultaneous causation between adolescent cigarette/alcohol use and premarital sex during college. After controlling for several other relevant variables in the model, our research was able to document the importance of substance use in predicting premarital sex during college. Secondly, in a Taiwanese context, our results may suffer from under-reporting of sexual behavior by youth due to the social desirability of sexual abstinence [42]. However, it should be noted extensive efforts were made in two stages to retain samples in the process. At the first stage, original samples (from Wave 1) were sent a notice of a follow-up survey. Each sample was then visited at least 3 times in order to conduct the interview at the field site. For participants who did not have access to or failed to provide consent for an interview, a second stage effort was involved. The district supervisor re-visited these samples’ family to gain access for interview. Since the survey began in 2000, New Year’s holiday greeting cards have been mailed annually to participants to maintain some contact. These efforts to generate long-term cooperative relationship with the participants since early adolescence helped to gain their trust, with less than 3% providing inconsistent answers regarding self-reported sexual experiences in the two surveys [8].

## Conclusions

In sum, our analyses clearly highlight gender differences in the temporal relationship between adolescent cigarette/alcohol use, perceived peer characteristics, stressful life and behavioral events, and the likelihood of engaging in premarital sex among Taiwanese college youth. Whereas group differences show higher proportions of cigarette and alcohol use among the youth who engaged in premarital sex than those who were sexually abstinent for both sexes, the multivariate analysis in the sexually abstinent group further indicates that two years later, adolescent cigarette smoking *decreased* as a predictor of subsequent premarital sex for females, while the effect of alcohol use remained a significant predictor of premarital sex. Reducing drinking, providing immediate counseling for those who experience a personal stressful event, and promoting positive attitudes toward intimate relationship before marriage during adolescents, may be helpful intervention message content in delaying the age of onset of premarital sex in young adulthood. Specifically, interventions promoting the delay of the onset of drinking and delivering appropriate sexual education during high school may be particularly useful in preventing Taiwanese college students from engaging in risky sexual behavior.

## Note

TYP data are released to public use and can be applied for research use by approval of Academia Sinica in Taiwan (http://www.typ.sinica.edu.tw).

## Competing interests

The authors declare that they have no competing interests.

## Authors’ contributions

CC was responsible for development of study hypotheses, data analysis, and drafting of the article. CCY and KK contributed to critical revision of the article. All authors involved in the writing of the paper, and all approved the final submission. All authors read and approved the final manuscript.

## Pre-publication history

The pre-publication history for this paper can be accessed here:

http://www.biomedcentral.com/1471-2458/12/527/prepub

## References

[B1] Chen CY Storr CL Tang GM Early alcohol experiences and adolescent mental health: A population-based study in Taiwan Drug Alcohol Depen 2008 95 209 218 10.1016/j.drugalcdep.2008.01.018 18342459

[B2] Hallfors DD Waller MW Bauer D Which comes first in adolescence – sex and drugs or depression? Am J Prev Med 2005 29 163 170 10.1016/j.amepre.2005.06.002 16168864

[B3] Meier AM Adolescent first sex and subsequent mental health Am J Sociol 2007 112 1811 1847 10.1086/512708

[B4] O'Donnell BL O'Donnell CR Stueve A Early sexual initiation and subsequent sex-related risks among urban minority youth: The reach for health study Fam Plann Perspect 2001 33 268 275 10.2307/3030194 11804436

[B5] Wen CP Levy DT Cheng TY Smoking behavior in Taiwan, 2001 Tob Control 2005 14 i51 i55 10.1136/tc.2004.008011 15923450 PMC1766183

[B6] Wen CP Chen T Tsai YY Are marketing campaigns in Taiwan by foreign tobacco companies targeting young smokers? Tob Control 2005 14 i38 i44 10.1136/tc.2004.007971 15923447 PMC1766177

[B7] Zabin LS Emerson MR Nan L Levels of changes in adolescent sexual behavior in three Asian cities Stud Family Plann 2009 40 1 12 10.1111/j.1728-4465.2009.00182.x 19397181

[B8] Chiao C Yi CC Adolescent premarital sex and health outcomes among Taiwanese youth: perception of best friends’ sexual behavior and the contextual effect AIDS Care 2011 23 1083 1092 10.1080/09540121.2011.555737 21562995

[B9] Jessor R Jessor SL Jessor R, Jessor SL The socio-psychological framework Problem behavior and psychosocial development: A longitudinal study of youth 1977 Academic, New York 17 42

[B10] Ortiz-Hernandez L Tello BLG Valdez J The association of sexual orientation with self-rated health, and cigarette and alcohol use in Mexican adolescents and youths Soc Sci Med 2009 69 85 83 10.1016/j.socscimed.2009.03.028 19427728

[B11] Sandfort TGM Orr M Hirsch JS Long-term health correlates of timing of sexual debut: Results from a national US study Am J Public Health 2008 98 155 161 10.2105/AJPH.2006.097444 18048793 PMC2156059

[B12] Mason WA Hitch JE Kosterman R Growth in adolescent delinquency and alcohol use in relation to young adult crime, alcohol use disorders, and risky sex: a comparison of youth from low- versus middle-income backgrounds J Child Psychol Psyc 2010 51 1377 1385 10.1111/j.1469-7610.2010.02292.x PMC2980793 20659188

[B13] Ajzen I Kuhl J, Beckmann J From intentions to actions: A theory of planned behavior Action control: From cognition to behavior 1985 Springer, Berlin, Heidelber, New York

[B14] Ajzen I The theory of planned behavior Organ Behav Hum Dec 1991 50 179 211 10.1016/0749-5978(91)90020-T

[B15] Ajzen I Perceived behavioral control, self-efficacy, locus of control, and the theory of planned behavior J Appl Soc Psychol 2002 32 665 683 10.1111/j.1559-1816.2002.tb00236.x

[B16] Akers RL Krohn MD Lanza-Kaduce L Social learning and deviant behavior: a specific test of a general theory Am Sociol Rev 1979 44 636 655 10.2307/2094592 389120

[B17] Bandura A Social learning theory 1977 Prentice-Hall, Englewood Cliffs, NJ

[B18] Akers RL Lee G Age, social learning, and social bonding in adolescent substance use Deviant Behav 1999 19 1 25

[B19] Upadhyay UD Hindin MJ Do perceptions of friends' behaviors affect age at first sex? Evidence from Cebu, Philippines J Adolescent Health 2006 39 570 577 10.1016/j.jadohealth.2006.03.004 16982393

[B20] Yang KS Kim U, Yang KS, Hwang KK Indigenous personality research: the Chinese case Indigenous and cultural psychology: understanding people in context 2006 Springer, New York 285 314

[B21] Huang FM Yi CC Yang JC Educational tracking, high school employment and college entry in Taiwan 2008 Paper Presented at the International Conference on Youth Studies, Academia Sinica, Taipei, Taiwan

[B22] Yi CC Wu CY Salter JJ Teen life in Taiwan Teen life around the world 2004 Greenwood Press, USA 223 241

[B23] Yi CC Wu CI Chang YH Chang MY The psychological well-being of Taiwanese youth: School versus family context from early to late adolescence Int Sociol 2009 24 387 429

[B24] Kaplan PS Adolescence 2004 Houghton Mifflin, Boston

[B25] Derogatis LR SCL-90-R administration, scoring, and procedures manual—II 1983 Clinical Psychometric Research, Towson, MD

[B26] Reiss IL The scaling of premarital sexual permissiveness J Marriage Fam 1964 26 188 197 10.2307/349726

[B27] Chiao C Yi CC Premarital sexual permissiveness among Taiwanese youth. In Yi CC. (ed) The psychological well-being of East Asian youth Springer, In Press, New York

[B28] Stata Corporation Stata statistical software: release 11 (Version Release 11) 2009 Stata Press, College Station, Tex

[B29] Morrison D Gillmore MR Hoppe MJ Adolescent drinking and sex Perspect Sex Repro H 2003 35 162 168 10.1363/3516203 12941648

[B30] Wells BE Kelly BC Golub SA Patterns of alcohol consumption and sexual behavior among young adults in nightclubs Am J Drug Alcohol Ab 2010 36 39 45 10.3109/00952990903544836 PMC5824634 20141395

[B31] Biglan A Metzler CW Wirt R Social and behavioral factors associated with high-risk sexual behavior among adolescents J Behav Med 1990 13 245 261 10.1007/BF00846833 2213868

[B32] Duncan SC Strycker LA Duncan TE Exploring associations in developmental trends of adolescent substance use and risky sexual behavior in a high-risk population J Behav Med 1999 22 21 34 10.1023/A:1018795417956 10196727

[B33] Chiao C Community context and the prevalence of premarital sex among young women in Kenya and the Philippines: Trends and differences from 1993 to 2003 Health Place 2010 16 512 522 10.1016/j.healthplace.2009.12.009 20089439

[B34] Chiao C Mishra V Trends in primary and secondary abstinence among Kenyan youth AIDS Care 2009 21 881 892 10.1080/09540120802537856 20024745

[B35] Gregson S Terceira N Mushati P Community group participation: can it help young women to avoid HIV? An exploratory study of social capital and school education in rural Zimbabwe Soc Sci Med 2004 58 2119 2132 10.1016/j.socscimed.2003.09.001 15047071

[B36] Adhikari R Tamang J Premarital sexual behavior among male college students of Kathmandu Nepal. BMC Public Health 2009 9 241 10.1186/1471-2458-9-241 PMC2717085 19604383

[B37] Ajuwon AJ Olaleye A Faromoju B Sexual behavior and experience of sexual coercion among secondary school students in three states in North Eastern Nigeria BMC Public Health 2006 6 310 10.1186/1471-2458-6-310 17187685 PMC1764888

[B38] Laub JH Sampson RJ Turning points in the life course: Why change matters to the study of crime Criminology 1993 31 301 326 10.1111/j.1745-9125.1993.tb01132.x

[B39] Brady SS Dolcini MM Harper GW Supportive friendships moderate the association between stressful life events and sexual risk taking among African American adolescents Health Psychol 2009 28 238 248 19290716 10.1037/a0013240 PMC2657930

[B40] Cooper ML Shapiro CM Powers AM Motivation for sex and risky sexual behavior among adolescents and young adults: A functional perspective J Pers Soc Psychol 1998 75 1528 1558 9914665 10.1037//0022-3514.75.6.1528

[B41] Rashad I Kaestner R Teenage sex, drugs and alcohol use: problems identifying the cause of risky behaviors J Health Econ 2004 23 493 503 10.1016/j.jhealeco.2003.09.009 15120467

[B42] Mensch BS Hewett PC Erulkar AS The reporting of sensitive behavior by adolescents: a methodological experiment in Kenya Demography 2003 40 247 268 10.1353/dem.2003.0017 12846131

